# Principles of Human Physiology, with Their Chief Applications to Pathology, Hygiène, and Forensic Medicine; Especially Designed for the Use of Students

**Published:** 1842-04

**Authors:** 


### Art. XIV.

*Principles of Human Physiology, with their chief applications to Pathology, Hygiène, and Forensic Medicine; especially designed for the Use of Students.* By William B. Carpenter, m.d.—*London,* 1842. 8vo, pp. 680.

Some of our readers may feel inclined to enquire, if after the publication of the native works of Dr. Bostock and of Dr. Alison, on Human Physiology, and after the appearance of English translations of the systematic treatises of Magendie, Müller, and Wagner, another systematic work on Human Physiology is really required? We deem it necessary therefore to settle this question before proceeding to the consideration of the contents of the volume before us.

The work of Dr. Bostock, though highly creditable to its learned author, and constituting a great boon to the medical profession at the time of its first publication, is now evidently lagging behind the rapidly increasing progress of physiological science, and many of its chapters would require to be entirely recast and rewritten to bring it up to the present state of our knowledge. Besides, in many parts of this work we find the authorities of so many authors pitched against each other, and conflicting opinions so nearly balanced, that the reader is quite at a loss to determine in what direction the truth is likely to be found.

The work of Dr. Alison is of a character entirely different from the preceding. We have here no poising of contradictory statements to distract the reader; but it consists essentially of announcements of the general principles which may be deduced from the best-established facts in this department of medical science, followed by brief statements of the most important data upon which these generalizations rest. We have on several occasions strongly expressed the high admiration we entertain of the labours of this philosophic physiologist, in whom extensive information and sound and vigorous judgment are harmoniously intermingled. However admirably fitted the "Outlines" of Dr. Alison are for his own students, for whom they were intended as a text-book, who enjoy the advantage of hearing fully detailed the different facts by which the generalizations at which he has arrived are confirmed and illustrated; or however useful they may be to those deeply read in physiology, to whom the various facts of the science are already known, they are too meager in their detail of facts to render them sufficiently acceptable to the generality of other readers.—Magendie appears to be so engrossed with his own individual exertions that he has neglected to avail himself of those of his contemporaries, so that though some parts of his work reflect high honour upon him as a successful labourer in experimental physiology, yet his "Compendium," when viewed as a whole, is meager and imperfect. The "Elements of Physiology" of Müller is a work of the very highest merit, and Dr. Baly has conferred a great benefit upon his professional countrymen by rendering its contents more generally known through his excellent translation. Müller has fully proved that he is not only intimately acquainted with the labours of others, but that his numerous successful exertions as an original investigator, justly place him in the foremost rank of distinguished living physiologists. Though every one who makes physiology an object of special study, must feel a delight in perusing and reperusing the pages of Müller, yet we know that the minuteness of many of their details,—take those of the organ of voice as an example,—are apt to damp the rising enthusiasm of the young student, and are totally unsuited for those who, chiefly bent upon practical information, wish to obtain a knowledge of the most important facts and principles in physiology without being obliged to wade through long and minute details, many of which *at present* do not admit of any practical application.—As one part only of the translation of Wagner's work has already appeared, we are unwilling to speak decidedly regarding it; but we may be allowed to state that, though we entertain the highest opinion of its merits, and give all honour to its author for the many new and important facts which it contains, yet we are satisfied that from the length and minuteness of its details upon points which are of greater scientific than of practical value, it will be more highly prized by the professed physiologist, than by those whose predilections, or want of time, prevent them from making this particular branch of medicine an object of special study. Besides, we believe that the arrangement of the contents of this work is not the best adapted for facilitating the progress of the student of physiology, though perhaps as unexceptionable as any other in a philosophical point of view.

From what we have said it must be evident to our readers that we are fully of opinion that another work on Physiology might be produced, capable of being extremely useful,—a work containing the philosophic cast of the Outlines of Alison, with an amount of the detail of facts sufficient at the same time to enforce and illustrate the generalizations, and to relieve the mind from the exertion of contemplating a rapid succession of abstract truths,—a work in which the reader may find himself under the direction of an intelligent guide, who has well weighed and decided upon the value of the conflicting testimony of those who have preceded him, without being involved in the mazes of minute and lengthy details, nor marched and countermarched over the field of controversy, until at last he becomes perplexed and bewildered. It must not for a moment be supposed that in making these remarks, we wish to encourage anything like dogmatism and overweening self-conceit in authors or that we condemn the exhibition of doubt and diffidence, where there is no distinct road, and where the light is obscure; for against these we have at all times strongly and willingly raised our testimony. We are sufficiently convinced that though many most important facts and principles have been already fully ascertained in Human Physiology, yet much uncertainty and confusion still exist on several very essential points, and that much labour and investigation are still necessary before it attains the correctness and precision of most of the physical sciences; and when we proceed to the elucidation of general principles, or to the details of individual functions, we have too frequent occasion to perceive the justice of the remark that " he who pretends to explain everything in Physiology, is either ignorant of his subject or of himself;" our meaning is that the author should, under the circumstances we have specified, write his work as much as possible in the form of a sustained narrative, without neglecting to express himself diffidently and cautiously when the subject requires it, and also taking care to inform his readers regarding the general character of the most plausible of the doctrines opposed to those which have been adopted by him after much thought and reflection. Neither let it be supposed that we adopt utilitarian views of science, and have any wish to depreciate the labours of those who minutely and accurately record facts, though at the time they may appear destitute of any practical bearing. We are perfectly aware that in the process of time, these for the most part, like isolated and detached recruits, will be ultimately incorporated each under an appropriate banner, and add strength to the onward progress of science; but we are satisfied that no one after a little reflection would maintain that they could be judiciously introduced into a work such as we are endeavouring to describe.

After a very careful examination of the contents of the volume before us, we were delighted to find that all the indications we have thrown out in the above remarks, are in its pages amply fulfilled, and that our author has provided us with a work admirably calculated not only to guide and direct the student of physiology, but from the agreeable mode in which old facts are presented, and new views opened up, also to afford pleasure and instruction to the deeply-learned in this branch of medical science. The author constantly endeavours to arrive at general principles; for he appears fully sensible that the science of physiology does not only consist in a systematic arrangement of the phenomena manifested by organized bodies, but also in classifying and comparing these "in such a manner as to deduce from them the general laws or principles by which they are regulated." Before any one, however, can successfully enter upon such an attempt, he must not only possess a vast store of facts and institute a careful comparison between them, but, above all, he must be deeply imbued with that cautious spirit of induction which enables him to decide when these facts are sufficiently numerous and authenticated to uphold the generalizations based upon them. We need not inform those of our readers who have perused our author's "Principles of General and Comparative Physiology," that his varied and extensive knowledge of facts, and his mental qualities admirably fit him for the task he has undertaken. We willingly record our hearty approval of the highly successful manner in which the following promise contained in the preface has been fulfilled:

> "On every topic it has been the author's aim to present the latest and most satisfactory information within his reach; and he believes that the volume contains much that will be new to the physiologist whose reading has not been tolerably extensive. Its materials have been but little derived from other systematic treatises on the subject; and it will not be found to bear, as a whole, any considerable resemblance to those already before the public. The author has rather endeavoured to bring together the valuable facts and principles scattered through the best of the numerous monographs that have been recently published on special divisions of physiology and medicine; and to reduce these *disjecta membra* to that systematic form, which they can only be rightly made to assume when brought into relation with each other, and shown to be subservient to principles of still higher generality." (p. 9.)

In collecting his facts, our author seems to have paid particular attention to the sources from which they have been derived, and to the value to be attached to each of them. This we have no hesitation in saying is one of the most important qualifications in a historian of science; and we cannot express the annoyance and regret we have sometimes experienced in finding in the works of some learned authors, authorities opposed and ranged against each other, which ought never to be put on the same level—an elaborate and carefully-conducted series of experiments, counterpoised by a few ill-observed and imperfect facts—the cautious induction of one who has made the subject an object of special study, neutralized by the unsupported assertion of another who has only cursorily glanced at it. He who collects together the opinions of authors ancient and modern, without at the same time searchingly scrutinizing the data upon which they formed their conclusions, and fixing their relative value, instead of being considered a benefactor to science, ought to be regarded not merely as a trifler, but almost as a nuisance, who stirs up the sediment of obsolete errors which was quietly subsiding, and renders everything as turbid as ever.

We have also had frequent occasions to lament the slavish deference shown by some authors to the opinions of those of great authority under circumstances where these illustrious persons themselves would have been the first to condemn. The opinions of such men as Hunter, Haller, &c.

are generally invaluable, and always deserving of the utmost consideration on those subjects which they have personally investigated; but it is undoubtedly absurd to place their opinions on other subjects, which they had not made an object of special study, and whose data they had not subjected to rigorous examination, against those drawn from a series of well-observed facts by other individuals, who, it may be, are far inferior in talent and reputation to those who have supported or advanced the opposite opinions. The authors of such works as we are now considering ought to endeavour not only to acquaint themselves with the name of the individual upon whose authority a fact or deduction rests, but also with the *means of knowledge*, which in that particular case he possessed. Were the whole science of medicine judiciously reexamined in this way, one of the greatest boons would be conferred on the medical profession; and there would then undoubtedly be infinitely less room for those numerous conflicting opinions which at present there is so much reason to deplore. Many of the doctrines which now perplex and divide medical men would be swept into everlasting oblivion. Such a task would, however, require the united labours of many dispassionate, industrious, and judicious individuals, and is not likely to be soon, if ever, accomplished.

In his introduction, the author, after pointing out very briefly the necessity "of bringing together all the phenomena of the same kind, in whatever class they may be present before we erect any general principles in physiology," goes on to state that "the objects of the present work is not to follow out such an investigation, but to show the detailed application of the principles of which physiological science may be said to consist, to the phenomena exhibited by the human being, during what may be called his normal life." Though he, therefore, restricts himself principally to human physiology, and with the view of rendering this knowledge more available in the practice of our profession, yet he justly never scruples to have recourse to comparative and even vegetable physiology when he wishes to illustrate and inculcate some great physiological principle, or to simplify and facilitate our knowledge of some particular facts. As Gerber remarks, "if we be made but a little lower than the angels, we are also very certainly made but a little higher than the more perfect among the animals; and in studying the structure and functions of these especially, we find the most important aids to a right understanding of the mechanism by which we ourselves 'live and move and have our being,' and thence, under the guidance of reason and experience, of the means by which we may hope to ward off or to remedy the ills in the shape of infirmity and disease to which we are made obnoxious."

The greater part of Dr. Carpenter's introduction is occupied with some judicious and clear remarks upon the connexion of physiology with other branches of medicine, and it is satisfactorily explained how every new important physiological discovery must put additional means in our power for detecting and curing some of those frequent deviations of the functions of the body from the healthy standard constituting disease. The necessity of a knowledge both of the principles and chief phenomena of physiology to him who devotes himself to the elucidation of the science of pathology is well illustrated by several examples. The following quotation will show, however, that the author does not lose sight of the cautious manner in which general principles require to be applied in the practice of so difficult an art as that of medicine:

"In the mean time the practitioner must be content to follow a middle course. His aim must be to avoid, on the one hand, confiding too exclusively in general principles, however stable and comprehensive he may imagine them to be, until he is satisfied that he knows not only the principle itself, but the subordinate laws which regulate or modify its application to individual cases. Long after the highest laws of motion had been established by Newton, no astronomer could, on the faith of them, have predicted the situation of a planet with more than approximation to certainty; the law of attraction had to be applied in numberless modes not contemplated by its discoverer, before perfect accuracy could be attained. There is great danger, then, in the present state of the science of pathology, in trusting to principles which we may consider unassailable, as our sole guides in the practice of our art; and hence it is not always the *scientific* practitioner, as he is emphatically termed, who is the most successful in his treatment. On the other hand, to apply a particular mode of treatment to a particular set of symptoms, without enquiring into the cause of those symptoms, merely on account of its having been successful in some case that *appeared* analogous, is a mode of practice completely empirical. Yet such a plan is constantly being pursued. But as soon as we begin to enquire into the cause of the morbid action, and seek to remove or counteract this by the application of remedial means which experience has shown to be effectual for such an object, we are really acting to a certain extent on scientific principles. The recorded experience of ages, in its condensed form, must of necessity assume the appearance of general rules of practice; and it is in the application of these rules to individual cases, and in the distinction of those phenomena whose causes are subservient to them, from those which are beyond their pale and which require a mode of treatment altogether different, that the sagacity of the practitioner most displays itself. The *rational empiricism* which prevails in this country at the present day, is a mode of practice that may be regarded as best combining the advantages of scientific knowledge and of recorded experience. The value of *facts* as the only sure basis of general principles is duly appreciated; and yet there is no indisposition to make trial of such *principles* when announced, and to abide by them so far as they appear practically available. This is the only method in which the young practitioner can hope to succeed. The increased attention at present paid to diagnosis, will frequently enable him to determine the real nature of the malady, with much more precision than can be done by a man of age and experience, who has not kept pace with the progress of medical science; whilst the latter will have decidedly the advantage in the application of therapeutic means, especially in those obscure cases which require the tact that can only be acquired by long and attentive observation." (pp. 8-9.)

At the end of the introduction we find a few but very valuable remarks upon the *numerical method*, which cannot be too strongly impressed upon those who are busily engaged in collecting statistical information for regulating practical medicine. We contemplate great ultimate advantages from statistical information, and there are many important facts which can only be elucidated through its means, but as it is apparently the fate of every important doctrine to suffer from the indiscretion of some of its friends, so we find that from the misapplication of the facts thus acquired, "they have a tendency to lead to the substitution of empirical rules for scientific principles; and if too exclusively followed, will tend to the retardation of pathology. If the practitioner is led to reason thus on every particular instance, 'in nine tenths of the cases exhibiting these symptoms, such and such a treatment is successful; therefore I will adopt this treatment in the present one,' he is acting on a most grossly empirical system." It is obvious that it is necessary for him, before he can escape from this charge that he advance onwards, and endeavour to ascertain the individual circumstances which prevent it from being successful in *all* the cases.

The first chapter, extending to forty-two pages, constitutes a new feature in a work on human physiology; it is occupied in pointing out the place of man in the scale of being. With a view of ascertaining this more definitely, our author takes a short but clear and comprehensive survey of the distinction between plants and animals, and between the different classes of animals. This we regard as a highly proper introduction to the study of the physiology of man, as we are fully satisfied that he who confines his attention to the vital phenomena of man and the higher animals must necessarily always possess very limited and imperfect notions on many most important doctrines in physiology. In this chapter the author brings prominently before us the important physiological fact that the manifestations of activity in plants are confined to nutrition and reproduction, while to these, animals also add sensation and voluntary motion. The possession of sensation and voluntary motion necessitates the presence of particular organs superadded to those of nutrition, which are more or less complex according to the position of the animal in the scale, its habits, and the media in which it lives. As these superadded organs, which place the individual in relation with the external world, are only to be found in animals, it was upon this circumstance that Bichat based his famous classification of the functions of the human body into animal and organic, or into those which are peculiar to animals, and into those which are common to all organized bodies. In the lower organized vegetables and animals, there are no special organs for absorption, digestion, circulation, respiration, and secretion; but every part of the simple structures of these organized bodies can perform all these functions in the feeble manner adequate to the maintenance of their vitality. As we rise in the scale both of the vegetable and animal kingdoms, we find that these functions are more or less concentrated upon particular parts of the organism, and we have distinct organs for absorption, circulation, respiration, &c. The concentration of an organ must be carefully distinguished from simplicity of structure, for the lowest zoophyte has an infinitely less complex structure than man and the other vertebrata, though the organs in the latter undoubtedly present a very striking contrast to the simple zoophyte in regard to concentration. In the soft pulpy homogeneous cellular structure of the zoophyte, we have no separate nutritive and reproductive organs, but each individual part has the property of performing all the nutritive and reproductive functions, or, in other words, these functions are diffused over the whole substance of the animal. In the vertebrata, on the other hand, each of these functions, or rather each of the great divisions of these functions, is concentrated, and is performed by a separate and distinct organ, and by it only. As the manifestations of activity in the vertebrata are eminently more varied and vigorous, it becomes necessary that the duties performed by these organs be increased in a corresponding degree, necessitating a more complex apparatus; but however complicated any organ may become, the function is evidently not the less concentrated or specialized. Thus, the concentration of organs in the higher animals, conjoined with the linking of them together by the nervous system, for the purpose of harmonizing their actions, and of subjecting certain of them to the influence of sensation and volition, explains in a very satisfactory and philosophic manner how the different parts of the lower animals and the vegetables have as it were individual existence, how the various portions of the organism in the higher animals are mutually dependent upon each other, and how the mutilation of one organ in the human body should so seriously affect all the remaining organs. A general statement of this kind, without an accurate conception of the important points of difference between the lower organized vegetables and animals on the one hand, and the higher organized animals on the other, to which we have just alluded, fails to convey the same clear conviction of its truth, and stands more in the relation of an isolated fact, which can never carry with it the same definite information, nor excite the same interest, as when the causes upon which the fact itself rests are at the same time present in the mind. When, for example, a slip of a plant or a portion of a polype are cut off, these, though small parts of particular organisms, are yet enabled, as we can easily conceive, to maintain an independent existence, for, from the diffusion of organs, each of these parts possesses the properties of reproduction and of nutrition, including both assimilation and respiration; while, when we proceed to the higher animals, a moment's reflection will convince us that it is impossible to select any particular part which is capable of performing these nutritive functions, without which it is obvious no organic structure can exist.

The author explains at some length the chief characteristic structural differences between man and such animals as the chimpanzee and orang-outan, which most nearly approach him; and in pointing out the admirable mechanism of the superior extremities, more especially of the hands, as organs of prehension, he presents us with some interesting remarks, which we shall partly quote:

"There is in him what we observe in none of the mammalia that approach him in other respects, a complete distinction in the functional character of the anterior and posterior extremities; the former being adapted for prehension alone, and the latter for support alone. Thus each function is performed with a much higher degree of perfection, than it can be where two such opposite purposes have to be united. The arm of the ape has as wide a range of motion as in man, as far as its articulation is concerned; but it is only when the animal is in the erect attitude that its arm can have free play. Thus the structure of the whole frame must conform to that of the hand, and must act with reference to it. But it cannot be said with truth (as some have maintained), that man owes his superiority to his hand alone; for without the directing mind, the hand would be comparatively valueless. His elevated position is due to his mind and its instrument conjointly; for if destitute of either, mankind would be speedily extinguished altogether, or reduced to a very subordinate kind of existence.......... Man cannot be regarded as distinguished from other mammalia, however, either by acuteness of sensibility, or by muscular power. His swiftness in running and agility in leaping are inferior to that of other animals of his size—the full-grown orang, for example......... He is surpassed by many in the acuteness of his sensibility to light, sound, &c.; but he stands alone in the power of *comparing* his sensations, and of drawing conclusions from them.......... Though his reasoning powers seem to differ rather in degree than in kind from those of the inferior animals, he seems distinguished by one innate tendency, to which we have no reason to suppose that anything analagous elsewhere exists. The tendency here referred to is that which seems universal in man, to believe in some unseen existence."

In his second chapter, which occupies twenty-three pages, our author very judiciously gives a general and connected view of all the functions of the body. This we consider a most important improvement upon the plan generally followed in physiological works, and must serve as a most suitable preparation for enabling the student to enter with advantage upon the examination of the details of individual functions. The functions of the human body are so interwoven together, and exert upon each other such reciprocal influence, that there is no one function in the body which is not dependent, to a greater or less extent, upon some others, and which does not, in its turn, react upon those by which it is itself influenced. There is no prime mover in the human body, as in a physical machine, from which we can set out and explain all the movements which follow. While some of the functions of an organ have a reference to itself only, there are others which are intimately connected with the manifestations of activity in other organs which at first sight appear the most removed and independent. Before the function of respiration can be effected in the human species and in the higher animals, it is necessary that the blood be driven to the lungs by the muscular contractions of the heart, and that air be drawn into the interior of the lungs by the contractions of certain muscles which dilate the thorax; but neither the excito-motory functions of the nervous system, through which the muscles of inspiration are called into action, nor the muscular contractions themselves can go on without the constant exposure of the blood to the atmospheric air. The movements of the heart, and the contractions of the other muscles of the body, are again intimately dependent upon fresh supplies of properly arterialized and elaborated blood, and these conditions can only be fulfilled by the reception of constant supplies of fresh materials from without. The first part of the process of deglutition in the adult is again performed through the intervention of volition and sensation; and before the food conveyed into the stomach can pass through the preparatory stage of assimilation which it there undergoes, certain fluids must first be eliminated from the blood by the manifestation of the function of secretion. It is obvious, therefore, that in the state of life, the organs cannot be considered as simply approximated, nor can they be described separately as in anatomical demonstration, but their actions are often blended together in effecting a common purpose. It is this mutual dependence of the functions, and the reciprocal assistance which they give and receive from each other, which demands that there should be a proper harmony between the organization of the structures which are thus mutually influenced, without which their vital activity could not be maintained; for if a single one of the functions was modified in a manner incompatible with the condition of the others, it is apparent that the animal could never live. A preparatory knowledge of this kind enables the student, when examining individual functions, to perceive the bearing of facts upon each other, and to recognize the importance of particular details, which would otherwise pass unnoticed.

In this general view of the functions of the body, our author has with his usual judgment taken, as the basis of his observations, the two great natural divisions into which the function of man, and all animals raised above the lowest in the scale, may be readily arranged, to which we have already had occasion to allude, viz. unto those by which the organism is built up and maintained, and the continuation of the species provided for ; and into those which place the organism in relation with the objects of the external world, and enable it to avoid what is hurtful and approach what is salutary. The possession by animals of an internal and complicated canal, surrounded by muscular fibres, the presence of a powerful muscular propelling organ in the circulatory apparatus, and the extensive muscular movements induced through the nervous system, by which air is drawn into the interior of the respiratory apparatus, and again expelled, do not, as our author judiciously points out, constitute any fundamental difference between the nutritive functions of animals and plants. The first is necessitated to place that portion of the nutritive in relation with the locomotive functions of the animal, and to furnish that more abundant supply of elaborated nutritious juices always conjoined with the more active manifestation of the vital actions, and the two latter to secure still farther the more rapid elaboration and purification of the nutritious juices, and an abundant and certain circulation of these through the different tissues. The muscular movements of the heart and intestinal canal habitually go on without our consciousness and volition ; those of respiration may also proceed without the intervention of sensation and volition, so that they are essentially organic functions.

"Thus we see that, when we enter, as it were, into the *penetralia* of the animal system, and study those processes of which the *life* of the material fabric consists, we find them performed under conditions essentially the same as those which obtain in plants; and we observe that the operations of the nervous system have none but an indirect influence or control over them. It is therefore quite philosophical to distinguish these *organic functions* or phenomena of vegetative life, from those concerned in the life of relation or animal life. The distinction is indeed of great practical importance, and lies at the foundation of all physiological science, yet is seldom accurately made, and a very confused notion on the subject is generally prevalent. It is commonly said, for example, that the function of respiration is the connecting link between the two: the fact being, however, that the true process of respiration is no more a function of animal life than is any ordinary process of secretion; but that, in order to secure that constant interchange of air which is necessary to its performance the assistance of the nervous and muscular systems is called in, though not in a manner which necessarily involves either *consciousness* or *will*." (p. 72.)

The distinction between these two sets of functions is still farther illustrated by the case "of no unfrequent occurrence, of a human being, deprived by some morbid condition of the brain, of all the powers of animal life—sensation, thought, volition, &c. ; and yet capable of maintaining a vegetative existence—all the organic functions going on as usual, the morbid condition not having affected that division of the nervous system which is concerned in the movements on which some of them depend." (p. 62.) Though we can assign no definite limits to such a state, as long as the necessary food is placed within reach of the grasp of the muscles of the back part of the mouth, yet, as our author remarks, "it is seldom of long continuance ; since the disordered state of the brain is sure to extend itself, sooner or later, to the rest of the nervous system." If the nutritive are, to a certain extent, dependent upon the animal functions, "the animal are even more closely dependent upon the nutritive actions; for many tissues will retain their several properties for a much longer period after a general interruption of the circulation, than will the nervous structure." (p. 62.) The function of reproduction in the animal and vegetable kingdoms is essentially the same in kind, and is only linked with the animal functions, even in the higher vertebrata, in the preliminary steps of the process, and to fulfil one of the conditions necessary in the propagation of all organized bodies where there is a division into sexes.

In his third chapter the author enters upon the consideration of the individual functions in detail, and very judiciously commences with the nervous system. He first gives a general summary of the functions of the nervous system, and, in doing so, presents us with the following sound and excellent remarks:

"It is well to explain that, though the physiologist speaks of the intellectual powers, moral feeling, &c. as *functions* of the nervous system, they are not so in the sense in which the term is employed in regard to other operations of the bodily frame. In general, by the *function* of an organ we understand some change which may be made evident to the senses, as well in our own system as in the body of another. Sensation, thought, emotion, and volition, however, are changes imperceptible to our senses by any means of observation we at present possess. We are cognisant of them in ourselves without the intervention of those processes by which we observe material changes external to our minds; but we judge of them in others only by inferences founded on the actions to which they give rise when compared with our own. When we speak of sensation, thought, emotion, or volition, therefore, as functions of the nervous system, we mean only that this system furnishes the conditions under which they take place in the living body." (p. 86.)

Many physiologists have attributed other functions to the nervous system besides those which our author enumerates,—such as the property of contractility, by which a muscle contracts upon the application of an excitant; and, to the presence of the nervous system, many also refer the property or susceptibility of the different tissues by which fresh materials are selected from the nutritious fluids in the performance of the function of nutrition. In the same manner many affirm that the different properties or susceptibilities of the various organs for secretion, by which they are enabled to eliminate such dissimilar compounds, as the bile, saliva, tears, gastric juice, &c., from the same fluid, the blood, depend upon the agency of the nervous system. Numerous facts are, however, adduced by Dr. Carpenter in different parts of his work, to prove that the nervous system is not necessarily connected with any of these functions, though each and all of these may be, and, in fact, in the natural and habitual condition of the body, really are, influenced in a most important manner through this system. Many of those who maintain that muscular contractility, that nutrition and secretion depend upon the nervous system, do not appear to appreciate fully the difference between the circumstances of a substance furnishing the conditions absolutely necessary for the performance of any action, and that when this action is only modified by the presence of this substance. To make use of an illustration which has already been used to inculcate this difference: we know that the movements of a horse are independent of the rider on his back, in other words, the rider does not furnish the condition necessary for the movement of the horse, as muscular movements, but every one is aware how much their movements may be influenced by the hand and heel of the rider. In the same manner we believe that muscular contractility, nutrition, and secretion are independent of the nervous system; yet we know that these may severally be increased, diminished, or even arrested by causes acting through the nervous system. Though we remove the property of muscular contractility, and the functions of nutrition, secretion, and absorption, from the list of those intimately and necessarily dependent upon the nervous system, we still leave many of the most important functions of an animal body under its entire control. In fact, the functions connected with this system are exactly those which distinguish the unconscious and passive vegetable from the active and reflecting mammal. The plant, though devoid of nerves, can assimilate the materials of the external world to its own structures, as man can do; it can eliminate various secretions from its nutritious juices as well as man. Several species of plants also exhibit undoubted evidence of contractility, as is well illustrated in the sensitive plant. It is only, however, in those animals raised above the lowest in the scale, and in whom the nervous system is somewhat developed, that the individual is enabled to become an active agent, to detect and avoid what is hurtful, and to search after and approach what is salutary. It is by the agency of the nervous system that all these combined actions are effected in the higher animals, by which the air is drawn into the lungs, and again expelled, as in the function of respiration, by which we are enabled to swallow, to articulate words, and to perform those numerous and varied manual operations, by which the sculptor and the painter are enabled to embody the finest and the noblest conceptions of the human mind upon the marble or the canvass, and to model and form all the numerous instruments and machines absolutely essential, not only for our humblest necessities, but without the aid of which there could be no science, and no arts. With the presence and integrity of the nervous system are also combined those manifestations of the intellectual faculties by which the various objects effected through the agency of the intellectual faculties, are conceived and planned. With the presence and integrity of the nervous system are also conjoined all those manifestations of the mental emotions and passions which agitate, afflict, and delight the human race.

After giving an excellent digest of the best-ascertained facts regarding the elementary structure of the nervous system, the author next presents us with a general view of the elementary functions of the nervous texture. The notion that the gray matter, from its great vascularity, and from its uniform presence in all those portions of the nervous system which manifest its peculiar functions, serves as the *source* of power; and that the white or medullary portions merely act as *conductors,* conveying changes from one part to another, has been, and we think justly, gaining ground of late, and this is the view adopted by our author. In the chapter on the functions of the cerebrum (p. 215) other arguments, drawn from pathological observations, are adduced in favour of this view. He adduces good grounds for believing that the *peripheral* as well as the *central* extremities of the *afferent nerves,* or those which convey inwards impressions to the central organs of the nervous system, are in close relation with a vascular plexus, and having a granular structure (as in the central substance of the brain,) intermediate between them. This is certainly the case in the peripheral expansion of the auditory and olfactory nerves, and in the papillæ of the skin and tongue.

"Our simplest idea of a nervous system includes a central organ, of which the gray matter formed by the intermixture of nervous fibres and blood-vessels, is the essential part; and an *afferent* and *efferent* set of fibres connected with it; one conveying to it the impressions produced by external changes upon the periphery (where also the nervous structure comes into peculiar relation with the vascular system), and the other conducting from it the motor stimulus originating in itself, to the contractile tissues."

The complex nervous system of man himself, when fairly scrutinized and analysed, is found to be essentially the same as this in kind, both anatomically and functionally.

Dr. Carpenter has, with great judgment, devoted a section to the consideration of the "mode of determining the functions of nerves," (p. 87;) a kind of knowledge, which we have looked upon as a great desideratum in books of this description. Every one who does not possess distinct and accurate information on the proper methods of ascertaining the different functions of the nerves must be constantly liable to fall into error in attempting to explain many phenomena which occur both in the healthy and diseased conditions of the system. Such a knowledge enables its possessor not only to scrutinize with confidence the inferences drawn from observations made on the functions of the nervous system either by himself or others, but suggests to him numerous sources of fallacy which would otherwise have escaped his notice. In a subsequent part of the work (p. 200) the author also points out the relative value of the results of negative and positive experiments in investigations into the functions of the nervous system—a distinction which is frequently overlooked, and cannot be too strongly impressed upon the mind. If a particular function be deranged, or even entirely disappear after the injury or destruction of a certain organ of the body, we cannot be quite certain that the performance of this function was connected with that organ, for the effects of the injury may have extended themselves to other organs, and not have been confined to that experimented upon. On the other hand, when we find a particular function, the performance of which is attributed to a certain organ remaining perfect after the complete destruction of that organ, then we have the most conclusive evidence that the performance of that function is not necessarily connected with the existence of that organ, for a thing cannot act where it is not. If, for example, we find the voluntary movements either imperfectly performed or arrested after destruction or the removal of the cerebellum, we are not certainly entitled to conclude that the function of volition is dependent upon the cerebellum, for the effects of incisions, hemorrhage, &c. necessarily attendant upon all such experiments, may affect the functions of other parts of the encephalon. Suppose, however, on the other hand, that the voluntary movements continue perfect after removal or complete destruction of the cerebellum, then we have the most conclusive of all evidence for affirming, that the functions of volition are not necessarily dependent upon the agency of the cerebellum, for it would be absurd to suppose that a function can be performed when the organ through which it is manifested is no longer in existence. It must also be sufficiently obvious that the deep incisions, hemorrhages, and infliction of pain, — the favorite topics of those who rail against *all* inferences drawn from experimental physiology,—cannot be urged against the results of the last experiment; on the contrary, these would all operate in favour of its accuracy; for if the function continued not only after the organ supposed to perform it had been removed, but also after various other injuries had been induced, it would afford, if this were possible, still stronger confirmation of the independence of that function of the presence of that particular organ. The former case, which we have here supposed, is termed a *negative* experiment, and must always be received with the utmost caution; the latter is a *positive* experiment, and is deserving of implicit confidence. One positive experiment will, when accurately performed, outweigh a whole host of negative experiments; yet how frequently do we find individuals ignorant of their relative value, cavilling at the inferences drawn from a positive experiment, and dogmatizing upon the results of a few negative experiments. When a negative experiment has been repeated several times, under varied circumstances, and invariably with the same results, and when we are also enabled, by positive experiment, to analyse the functions of the organ to a certain extent, and to ascertain what particular functions are not performed by it, sufficiently accurate data may be obtained to enable us to draw our conclusions with perfect accuracy. For example, it being previously ascertained that sensation and the voluntary movements depend upon the nervous system, if we then proceed to cut the portio dura nerve, and find that the sensation of the face remains quite perfect, though motion be lost, we are entitled to conclude, from this experiment, that the sensations of the face are not dependent upon this nerve, but we cannot be equally certain that the loss of voluntary motion over the muscles of expression of the face was essentially dependent upon the division of the nerve, since some other accidental circumstance may have induced this. If, after several repetitions of this experiment, this loss of voluntary motion be *invariably* produced, we may be pretty confident that these movements are connected with the integrity of this nerve, and this amounts to perfect conviction by experiments on the fifth pair, the only other nerve of the face distributed in these muscles, in which the very opposite results follow, viz. loss of common sensation and persistence of the voluntary movements.

We conceive that it is as much the duty of him who undertakes the guidance of those entering upon the study of physiology to endeavour to train them "to the high task of correctly interrogating and interpreting nature," as it is to furnish them with the facts and principles of the science. Our author, in directing the attention of his readers to those portions of the comparative anatomy and physiology of the nervous system, which assist us in unravelling the complicated functions of man, very justly remarks that "although the structure and distribution of the nervous system in the different classes of animals have been, until recently, but little appealed to in the determination of its functions, they are capable of supplying evidence regarding some of these not less important in its character than that which comparative anatomy affords to other departments of physiology." (p. 91.) As we had on a former occasion (vol. VIII. p. 506,) an opportunity of directing the attention of our readers to the results of the investigations of our author in this interesting and important department of physiology, contained in his "Prize Thesis," we shall simply content ourselves with an expression of our conviction of the great value of this part of the work to those entering upon the study of the more complex nervous system of man.

In his account of "the anatomy and functions of the nervous system," Dr. Carpenter has implicitly adopted both the doctrine of Sir Charles Bell, on the difference between the volitional and sensiferous and nervous filaments, and the excito-motory doctrine of Dr. Marshall Hall. The doctrine of Sir Charles has now fairly and successfully passed through the severe ordeal to which every important discovery is generally and justly subjected; that of Dr. M. Hall appears to be rapidly immerging from it. It is in the vertebrated animals that the difference between the sensiferous and volitional nerves has been satisfactorily determined, while "on the other hand, the distinctness of the system of nerves concerned in the simply-reflex actions, from those which minister to sensation and volition by their connexion with the brain, is by no means so obvious as in the invertebrated classes." (p. 118.)

An excellent general description of the "arrangement of the nervous system of man" appropriately follows that of the other animals beneath him in the scale, and mutual light is thrown upon them by contrast and comparison. The following passage will show the manner in which this is managed :

"There is no reason to believe that the functions of the spinal cord are essentially different along its whole length. Everywhere it appears to consist of a ganglionic centre, supplying nerves to its particular segment, and of connecting fibres, by which the nerves proceeding from any one division are brought into relation with distant portions of the organ, and with the large ganglionic masses at its anterior extremity. In this respect, then, it corresponds precisely with the double nervous cord of the articulata; the only prominent difference between the two being, that in the former the ganglionic matter is continuous from one extremity of the organ to the other, whilst in the latter it is interrupted at intervals; and in the mollusca the centres are still further separated from each other. The connexion of the spinal cord with the large ganglia contained within the cavity of the cranium is effected by means of processes from its superior extremity, the arrangement of which is somewhat complex. This portion of the cord, which also lies within the cavity of the cranium, has been termed the medulla oblongata. It has been supposed to be the peculiar seat of vitality; but the only real foundation of this idea is, that it is the great centre of respiratory actions, on the continuance of which all the other functions are dependent. The brain may be removed from above, and nearly the whole spinal cord from below, without an immediate check being put upon all the nervous phenomena of life." (p. 123.)

A general description of the "anatomy of the medulla oblongata" follows this.

Under the "functions of the spinal cord," the author describes, and at considerable length, all the excito-motory muscular movements, as those of respiration, deglutition, those which control the orifices of the various open cavities of the body, &c., and has availed himself of the results of all the late investigations into the functions of individual nerves, in explaining the particular afferent and efferent nerves engaged in the performance of these muscular movements. The various changes which take place between the blood and the atmospheric air, or the function of respiration, properly so called, are examined separately, and after the account of the circulation of the blood.

Dr. Carpenter has, in our opinion, very properly refused to class the peristaltic contractions of the intestines among the excito-motory movements, maintaining "that the intestinal tube from the stomach to the rectum *is not dependent* upon the spinal cord for its contractility, but is enabled to propel its contents by its own inherent powers." While, however, he contends that the muscular fibres of the intestines are excited to contraction by direct stimulation, he points out that "the nervous centres exert a general control over even the organic functions." (p. 151.) Before leaving the account of the principal functions of the spinal cord, he adverts to some of the leading pathological applications of the physiological doctrines which can be deduced from it, and illustrates the greatly increased precision which our knowledge of many convulsive and spasmodic diseases has acquired from the researches of Dr. Marshall Hall. (p. 159.) This important subject is again introduced at greater length at p. 227.

The author now proceeds to examine the "functions of the *encephalon;*" and, after taking a general view of the comparative anatomy of that portion of the central organs of the nervous system, he examines in detail the functions of the encephalic nerves. In this, as in every other part of the work, Dr. Carpenter exhibits his great knowledge of facts, and, what is of more importance, of their proper relation to each other. He has given an excellent account of the functions of the motor nerves of the orbit, and of the consensual movements of the muscles of the eyeball,—subjects which of late have attracted a good deal of attention in connexion with the operations for strabismus. After some very interesting remarks upon the "relation between *instinct* and *intelligence*," we find, at p. 198, a very ingenious attempt to localize the seat of those instinctive actions which are prompted by sensation: this we beg to lay before our readers.

"If the essential correspondence between the purely emotional acts of man, and the instinctive acts of the lower animals be admitted, we may reasonably localize the centre more satisfactorily in that chain of ganglionic masses which only occupies the centre of the base of the brain in man, but which in the lower vertebrata possesses an aggregate dimension far exceeding that of the cerebral hemispheres. We are led to such a localization by a very simple and satisfactory chain of reasoning. The actions in question are not simply reflex; since sensation and something of the nature of emotion, both involving consciousness, are elements in their performance; and, moreover, these sensations are rather of the *special* than of the *common* character, involving, therefore, the olfactory, optic, and auditory ganglia. No intelligent person can doubt that, as we descend the scale of being, instinct is gradually superseding reason; and that in the lowest vertebrata (we go no further, because no comparison between the parts of the nervous centres could be made with equal certainty between animals of a different type), the manifestations of the latter are extremely feeble, nearly all the actions of life being guided by the former. Now, on looking at the encephalon, we perceive a difference in the relative proportions of its principal divisions so closely corresponding with these, that it is difficult to imagine them unconnected. In proportion as we descend the scale, we find the cerebral hemispheres diminishing in relative size, whilst the ganglia at the origins of the nerves of special sensation increase to a remarkable degree; and we cannot, therefore, but consider it probable that these ganglia and tracts of gray matter, whose size is in man so trifling in comparison to the bulk of his cerebral hemispheres, are subservient to those instinctive actions which are prompted by sensations, but in which volition does not partake."

In examining the "functions of the cerebellum," our author, after remarking that the particular purposes which are served by this organ are still much in the dark, proceeds to detail a number of arguments drawn from comparative anatomy, from the result of experiments, and from pathology, in favour of the view that the faculty of combining the muscles in groups in those actions which are not excito-motory are dependent upon this portion of the encephalon. Though he has adduced a formidable array of facts in favour of this opinion, yet we confess that after a careful examination of all the evidence advanced by its supporters, we feel less inclined to admit this doctrine than he appears to do, and we would willingly break a lance with him on this point, did our time admit of it. As our space is already nearly exhausted, we must hasten onwards, and content ourselves by giving our readers a cursory glance of the contents of some other parts of the work. The remainder of this section of the volume is occupied with a detailed account of the "function of sensation, and the organs of the senses," to some parts of which we would willingly have directed the attention of our readers, had we not, from the great interest of the preceding part of the volume, lingered so long upon it.

After finishing the consideration of the nervous system, the author properly proceeds to the examination of the "intimate structure and functions of the muscular tissue, and the organs of the voice and speech," as they form the remaining parts of the apparatus of animal life. In describing the intimate structure of the muscular fibre, he has availed himself freely of the late researches of Mr. Bowman, as this gentleman's results are those most conformable to his own observations; and several representations of the appearance of the muscular fibre under the microscope are given to enable the reader fully to comprehend the description. In examining the functions of the muscular tissue, we are glad to find that our author maintains, on what we believe to be very sufficient grounds, that the property of muscular contractility is not derived from the nervous system, and has taken care to point out the insufficiency of the arguments of Müller and Dr. Marshall Hall in favour of the opposite doctrine. He is, however, at the same time careful to point out that, though muscular contractility is independent of the nervous system, yet it may be influenced to a most important extent by causes acting through the nervous system, as is well illustrated in the effects of extensive injuries of the brain or spinal cord upon the contractility of the heart, to which the term Concussion is applied. The contractions of the heart, if not altogether arrested, become weak and rapid, and the flow of blood through the capillaries is also enfeebled. We can, therefore, readily explain the weak, small, and rapid pulse, the cold extremities, the sighing respiration, and the cerebral derangement which form the prominent symptoms of concussion. If, on the other hand, the injury be confined to a smaller portion of the brain, the contractility of the heart may be little if at all affected, and the individual may be deprived of consciousness, and lie with dilated pupils, and slow and heaving respiration occurring at long intervals, presenting a condition generally termed *compression.* The contractility of the heart and the capillary circulation continuing comparatively unaffected, explain the persistence of the full pulse and warm skin, while the dilated pupil and slow heaving respiration depend upon the injury done to the optic lobes and medulla oblongata. We can readily understand how the extent of these latter symptoms should not have any fixed relation to the state of unconsciousness. Our author takes care to point out that the same debilitating effects upon the heart's action and capillary circulation may be induced by extensive burns of the surface of the body, by severe injuries of the extremities, as in compound fractures, by sudden and rapid peritonitis, &c. These are most important practical facts, which cannot be too deeply studied by all those who wish to acquire enlightened views of pathology.

Before proceeding to the consideration of the nutritive functions the author devotes a short chapter to the examination of the "influence of the nervous system on the organic functions," a subject of considerable importance both in a physiological and pathological point of view. The effects of the emotions of hope and joy, and the agreeable sensations, when not excessive and transient, but more moderate and steady, in promoting the capillary circulation, and encouraging the necessary secretions; and, on the other hand, the effects of the depressing passions, such as grief, anguish, and despair, in enfeebling the capillary circulation, diminishing or vitiating the secretions, and enfeebling the contractility of the heart, and thus favouring the influence of those causes which induce disease, and impede the operations of those actions by which the body may rid itself of its maladies,—are all clearly explained and illustrated.

In examining the nutritive functions in detail, our author proceeds in the most methodical manner he could adopt. He traces the course of the food from its first introduction into the alimentary canal, until it becomes intermixed with the juices already elaborated in the blood, and describes the various changes, as far as they are known, which it undergoes in its first process of assimilation in the stomach, and in its subsequent progress along the lacteals and thoracic duct. These details necessarily require him to enter into an account of the function of *absorption.* He is then led to the consideration of the mode in which the blood is circulated; and from this he naturally passes to the examination of the only remaining process engaged in elaborating the ingesta into a nutritious fluid fitted for maintaining the vital actions of the different tissues, viz., the function of respiration. Having thus examined the processes by which the plenitude of the blood-vessels is maintained, and the mode in which the fully-elaborated nutritious fluid, or the arterial blood, is circulated through the various structures of the body, he proceeds to describe, as far as they are known, the different molecular changes by which certain materials are eliminated from the blood, and deposited in the tissues for their nutrition; and also the processes by which other materials are separated from the blood by the organs of secretion. The only remaining process which can properly be included under the nutritive functions is that by which various materials which apparently cannot be converted into compounds fitted for the nutrition of the tissues, are separated from the blood and thrown out of the body, constituting the function of excretion. The author next takes a general review of the nutritive functions, and this is followed by an account of the process by which the animal heat is generated. The development of animal heat has, for a considerable time past, been generally described as part of the respiratory function, but there can be no doubt that this is due to the molecular changes of nutrition and secretion generally, of which those of the lungs form a part, and that our author is perfectly right in placing it where he has. The detailed account of these various processes necessarily occupies a considerable portion of the volume: in reading them, we marked out a number of the passages, most deserving of being quoted and commented on, but our circumscribed limits forbid this. The execution of this part of the work is equal to that which we have already gone over.

In the chapter on "digestion" we find a perspicuous and complete analysis of the experiments of Dr. Beaumont on this subject; also those of Eberle and Schwann on artificial digestion, and those of Wasmann upon *pepsin*, the active principle in the gastric juice. We have also an account of the results of the late interesting chemical analysis of fibrin, albumen, casein, &c., by Mulder and Liebig, which promise to throw much light on the process of *assimilation;* and among these our author has intermingled many ingenious comments, which we regret to be obliged to pass over without further notice. The chapter on digestion is closed with some valuable practical remarks upon the supply of food required by man.

In the chapter on the "circulation of the blood," we would particularly direct the attention of our readers to the observations upon the causes influencing the circulation in the arteries and capillaries, as we are fully convinced that the doctrines maintained by the author on this subject are correct, and that the facts there adduced afford a complete refutation of the notions most generally entertained on this subject,—opinions fraught with many grave errors. "The idea of any *mechanical* assistance, afforded by the action of the capillaries to the movements of the blood," must be got rid of before any steady advances can be made in some departments of pathology, for it directs research in a wrong track.

The chapter on "nutrition" is especially deserving of attention, as great advances have lately been made in our knowledge in this quarter, and our author has collected, from the scattered papers lately published, a connected and systematic view of all that is *at present* known on the subject, which the reader will in vain search for elsewhere. There is one passage here, with the truth of which we are forcibly struck, which we cannot avoid quoting:

"Several instances have been recently published of the occurrence of vegetable organisms as parasites upon the animal body: that in some of these a true plant, possessing a regular apparatus of nutrition and reproduction, has arisen from a germ introduced from without, there can be little question; but in other instances (as in the case of crusts of *porrigo favosa*) it has been assumed that the organization is vegetable, because it consists of a mass of cells capable of extending themselves by the ordinary process of multiplication. But it must be remembered that the vesicular organization is common to animals as well as to plants; being the only form that manifests itself at an early period of development in either kingdom, and remaining throughout life in those parts which have not undergone a metamorphosis for special purposes. Hence to speak of *porrigo favosa,* or any similar disease, as produced by the growth of a vegetable within the animal body, appears to the author a very arbitrary assumption; the simple fact being, in regard to this and many other structures of a low type, that they present the simplest or most general kind of organization." (p. 458.)

The volume is terminated by an account of the "function of reproduction." Our notions on many points of this process have, of late years, undergone a complete revolution, and many talented and industrious investigators are, at the present moment, engaged in unfolding its mysteries. Here, as in every other part of the work, we find our author quite *au courant* with the present state of our knowledge.

In reading over a work such as this, containing a great multiplicity of facts and inferences from them, it is impossible to suppose that we could give our free assent to all the statements which it contains, but we can most conscientiously declare that, after a careful examination of it, we could find nothing where we could with confidence affirm that the author was wrong, and we were right; and the few points on which we differ were of minor importance, and not worthy of being commented upon. When we say that the merits of this volume are equal to that of the author's "Principles of Comparative Anatomy and General Physiology," we are bestowing upon it very high praise. The style is everywhere easy, perspicuous and appropriate to the subject. The numerous woodcuts and engravings with which the descriptions are illustrated, are judiciously selected, and excellently executed. We speak advisedly when we say that we know of no work on physiology from which the student is likely to derive so much advantage: the whole of it reflects the highest honour upon the talents, knowledge, and judgment of the author.

---

